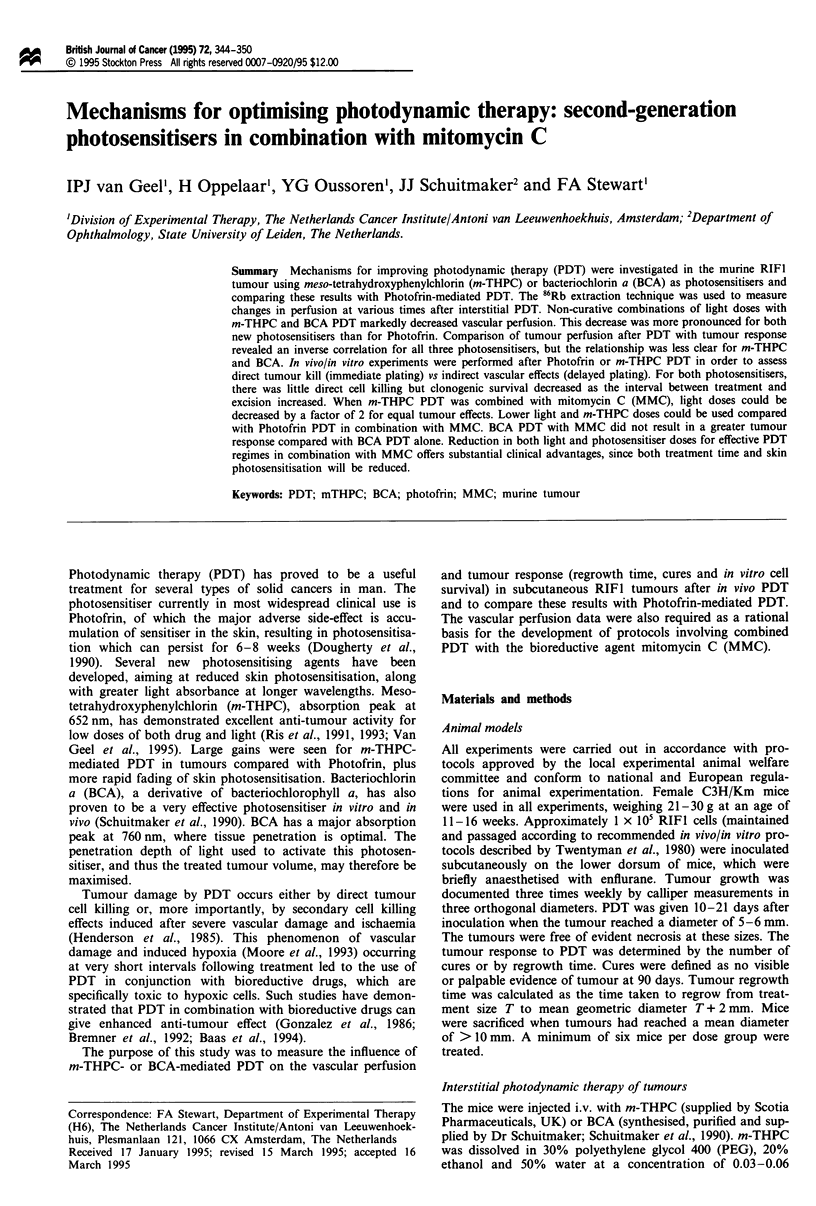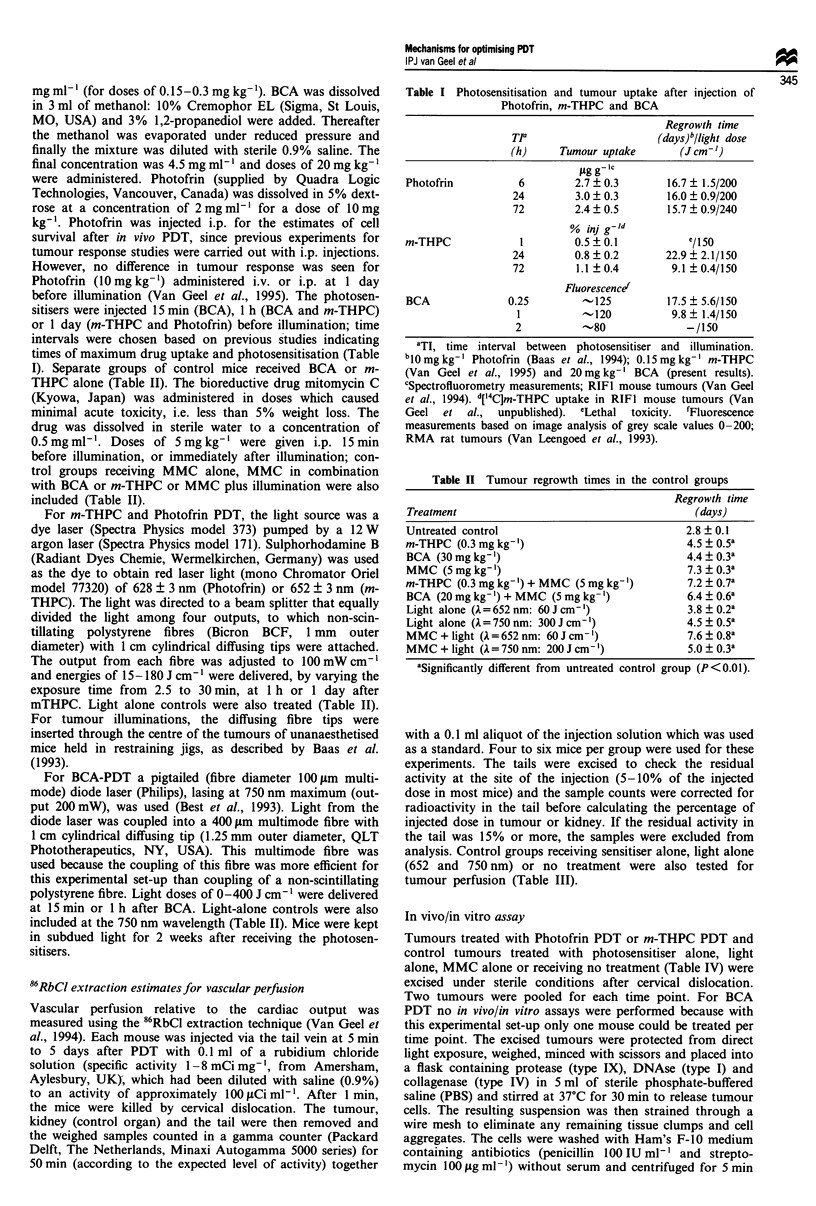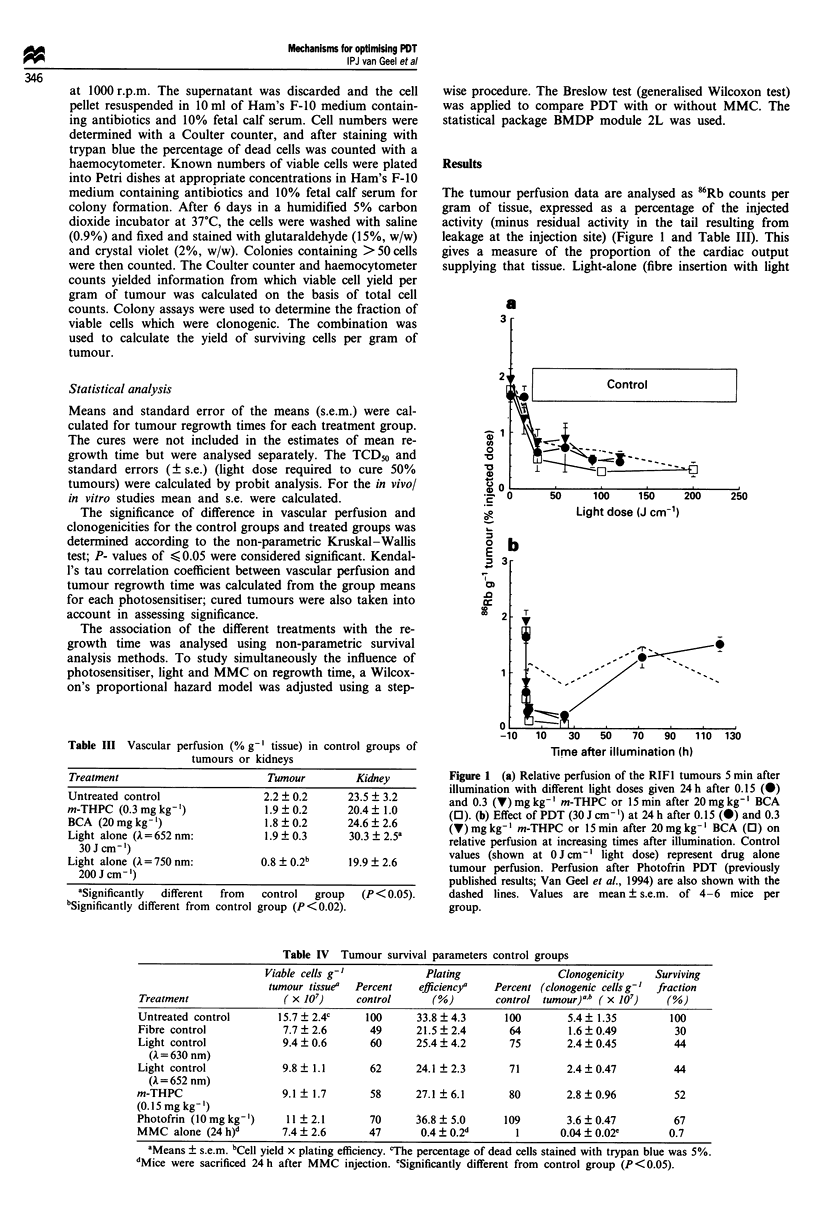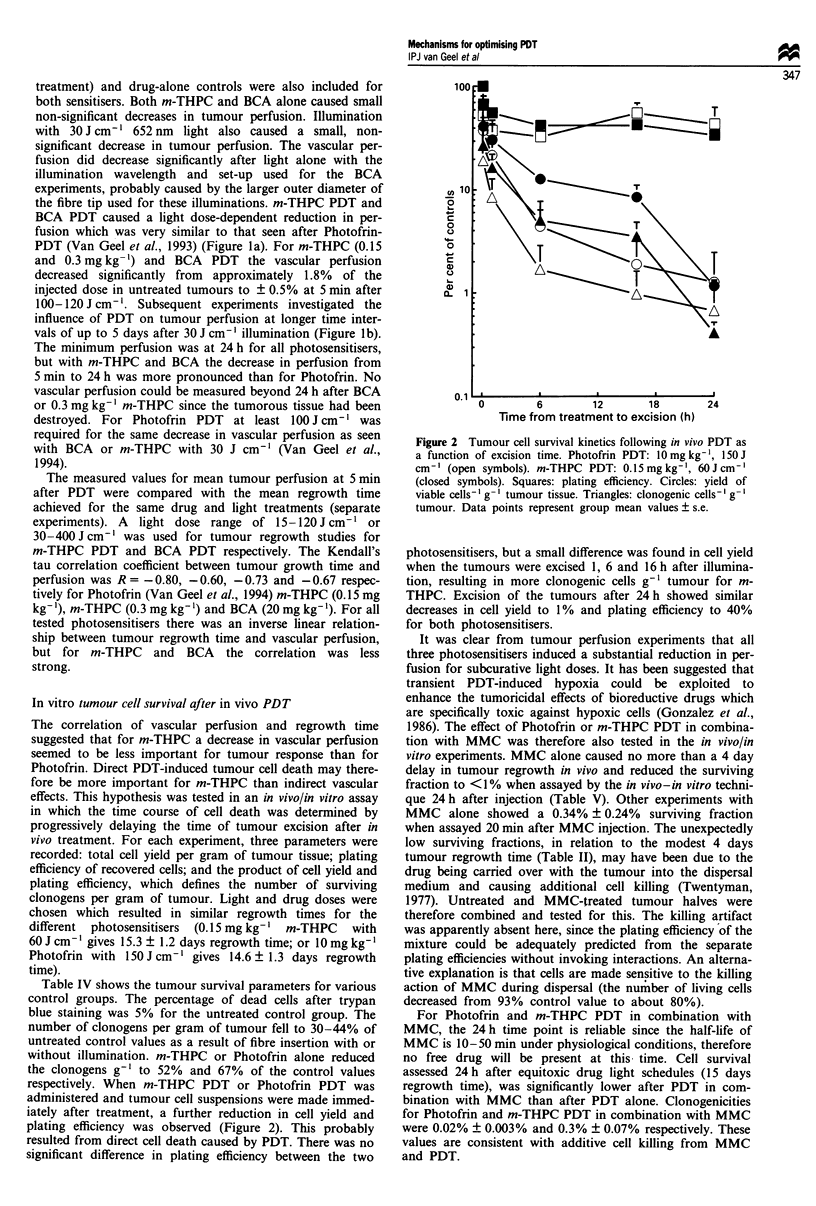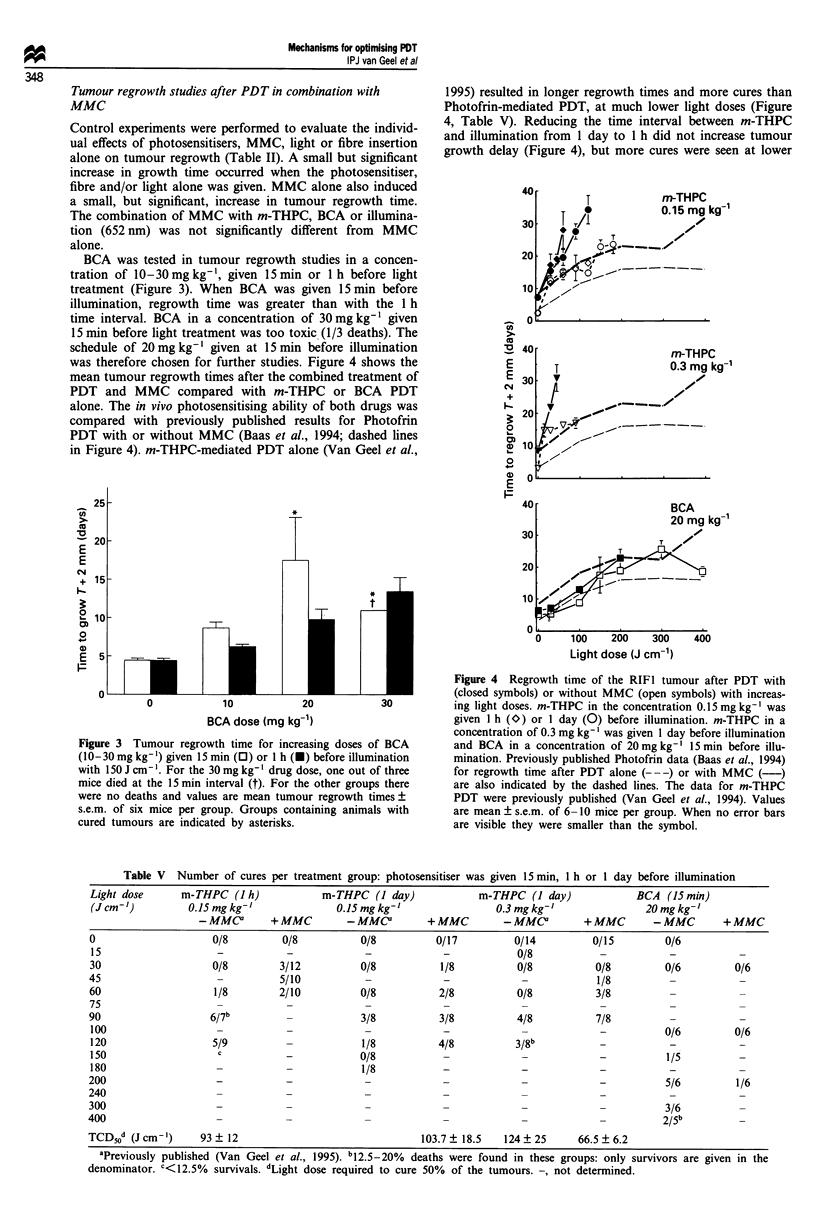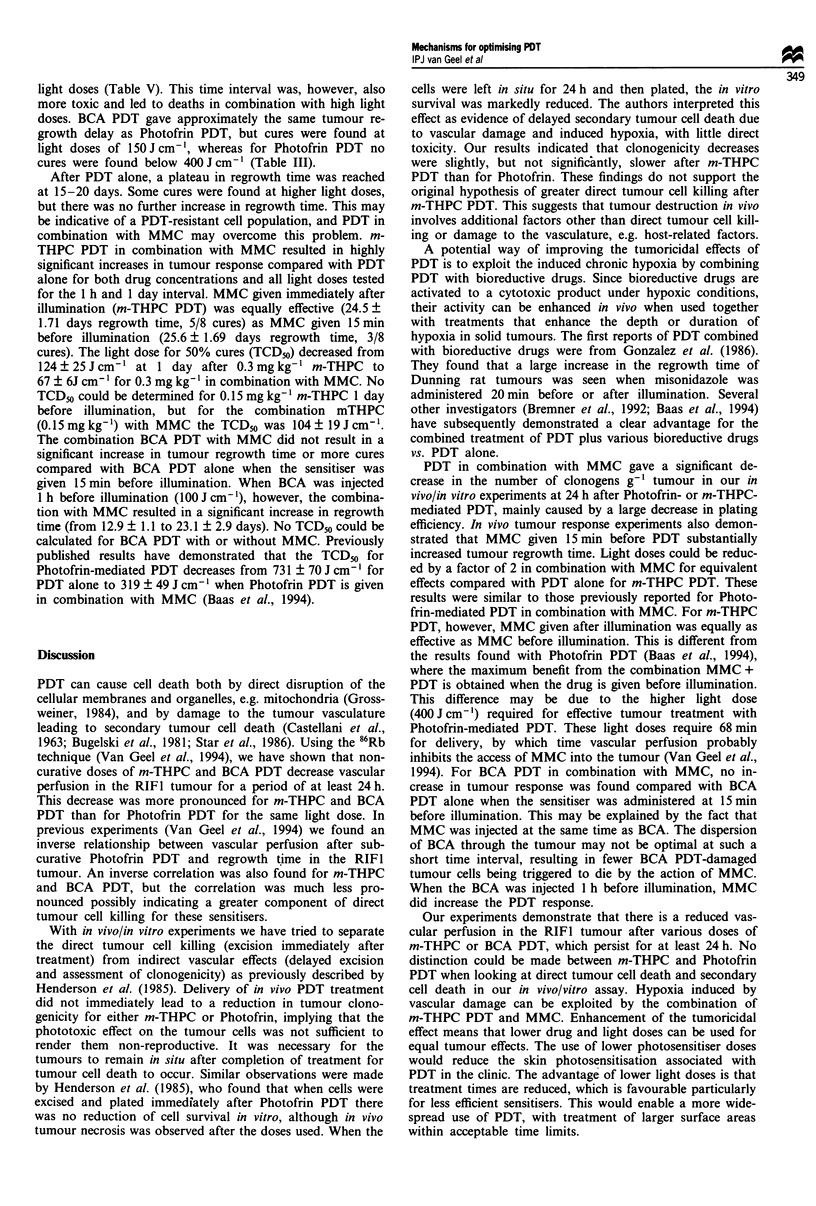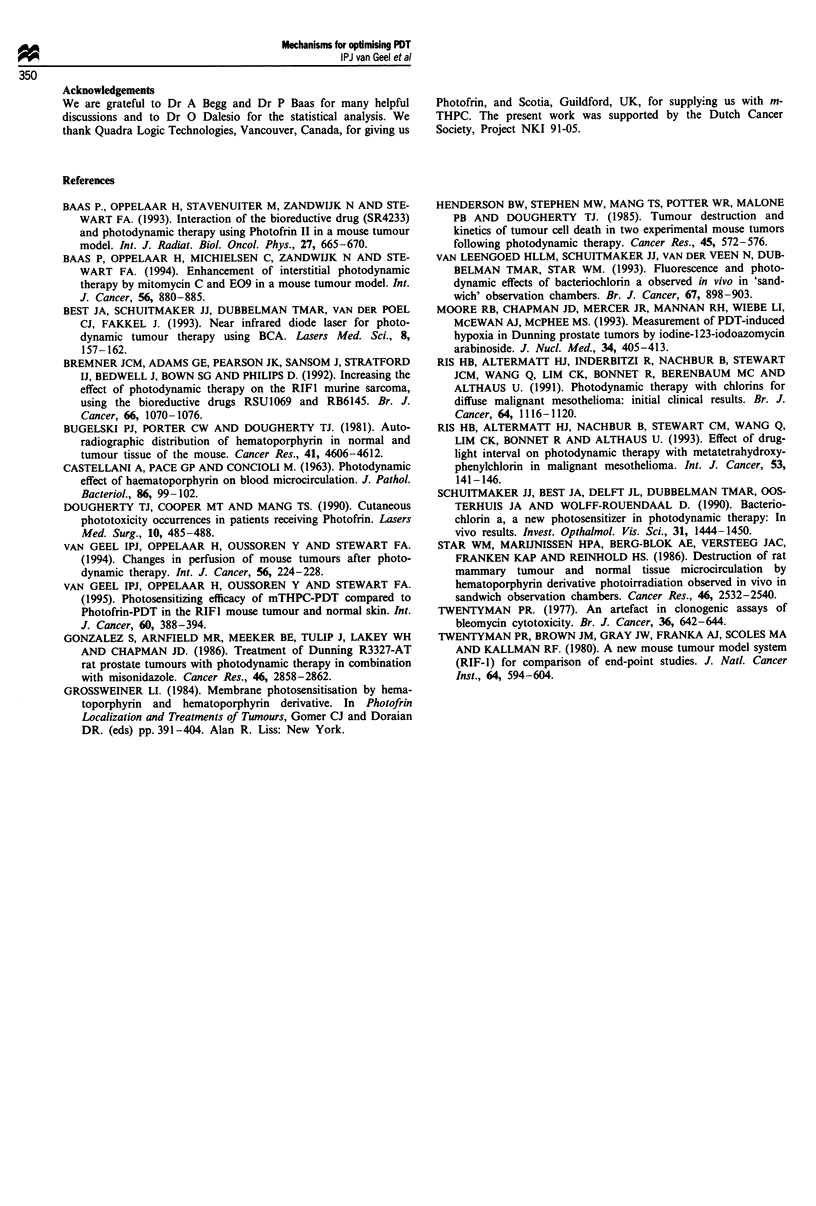# Mechanisms for optimising photodynamic therapy: second-generation photosensitisers in combination with mitomycin C.

**DOI:** 10.1038/bjc.1995.336

**Published:** 1995-08

**Authors:** I. P. van Geel, H. Oppelaar, Y. G. Oussoren, J. J. Schuitmaker, F. A. Stewart

**Affiliations:** Division of Experimental Therapy, The Netherlands Cancer Institute/Antoni van Leeuwenhoekhuis, Amsterdam.

## Abstract

Mechanisms for improving photodynamic therapy (PDT) were investigated in the murine RIF1 tumour using meso-tetrahydroxyphenylchlorin (m-THPC) or bacteriochlorin a (BCA) as photosensitisers and comparing these results with Photofrin-mediated PDT. The 86Rb extraction technique was used to measure changes in perfusion at various times after interstitial PDT. Non-curative combinations of light doses with m-THPC and BCA PDT markedly decreased vascular perfusion. This decrease was more pronounced for both new photosensitisers than for Photofrin. Comparison of tumour perfusion after PDT with tumour response revealed an inverse correlation for all three photosensitisers, but the relationship was less clear for m-THPC and BCA. In vivo/in vitro experiments were performed after Photofrin or m-THPC PDT in order to assess direct tumour kill (immediate plating) vs indirect vascular effects (delayed plating). For both photosensitisers, there was little direct cell killing but clonogenic survival decreased as the interval between treatment and excision increased. When m-THPC PDT was combined with mitomycin C (MMC), light doses could be decreased by a factor of 2 for equal tumour effects. Lower light and m-THPC doses could be used compared with Photofrin PDT in combination with MMC. BCA PDT with MMC did not result in a greater tumour response compared with BCA PDT alone. Reduction in both light and photosensitiser does for effective PDT regimes in combination with MMC offers substantial clinical advantages, since both treatment time and skin photosensitisation will be reduced.


British Journal of Cancer (1995) 72, 344-350
© 1995 Stockton Press All rights reserved 0007-0920/95 $12.00

### Mechanisms for optimising photodynamic therapy: second-generation photosensitisers in combination with mitomycin C

IPJ van Geel[1], H Oppelaar[1], YG Oussoren[1], JJ Schuitmaker[2] and FA Stewart[1]

[1]*Division of Experimental Therapy, The Netherlands Cancer Institute/Antoni van Leeuwenhoekhuis, Amsterdam;* [2]*Department of Ophthalmology, State University of Leiden, The Netherlands.*

**Summary** Mechanisms for improving photodynamic therapy (PDT) were investigated in the murine RIF1 tumour using *meso*-tetrahydroxyphenylchlorin (*m*-THPC) or bacteriochlorin *a* (BCA) as photosensitisers and comparing these results with Photofrin-mediated PDT. The $^{86}$Rb extraction technique was used to measure changes in perfusion at various times after interstitial PDT. Non-curative combinations of light doses with *m*-THPC and BCA PDT markedly decreased vascular perfusion. This decrease was more pronounced for both new photosensitisers than for Photofrin. Comparison of tumour perfusion after PDT with tumour response revealed an inverse correlation for all three photosensitisers, but the relationship was less clear for *m*-THPC and BCA. *In vivo/in vitro* experiments were performed after Photofrin or *m*-THPC PDT in order to assess direct tumour kill (immediate plating) *vs* indirect vascular effects (delayed plating). For both photosensitisers, there was little direct cell killing but clonogenic survival decreased as the interval between treatment and excision increased. When *m*-THPC PDT was combined with mitomycin C (MMC), light doses could be decreased by a factor of 2 for equal tumour effects. Lower light and *m*-THPC doses could be used compared with Photofrin PDT in combination with MMC. BCA PDT with MMC did not result in a greater tumour response compared with BCA PDT alone. Reduction in both light and photosensitiser doses for effective PDT regimes in combination with MMC offers substantial clinical advantages, since both treatment time and skin photosensitisation will be reduced.

**Keywords:** PDT; mTHPC; BCA; photofrin; MMC; murine tumour

Photodynamic therapy (PDT) has proved to be a useful treatment for several types of solid cancers in man. The photosensitiser currently in most widespread clinical use is Photofrin, of which the major adverse side-effect is accumulation of sensitiser in the skin, resulting in photosensitisation which can persist for 6-8 weeks (Dougherty *et al.*, 1990). Several new photosensitising agents have been developed, aiming at reduced skin photosensitisation, along with greater light absorbance at longer wavelengths. Meso-tetrahydroxyphenylchlorin (*m*-THPC), absorption peak at 652 nm, has demonstrated excellent anti-tumour activity for low doses of both drug and light (Ris *et al.*, 1991, 1993; Van Geel *et al.*, 1995). Large gains were seen for *m*-THPC-mediated PDT in tumours compared with Photofrin, plus more rapid fading of skin photosensitisation. Bacteriochlorin *a* (BCA), a derivative of bacteriochlorophyll *a*, has also proven to be a very effective photosensitiser *in vitro* and *in vivo* (Schuitmaker *et al.*, 1990). BCA has a major absorption peak at 760 nm, where tissue penetration is optimal. The penetration depth of light used to activate this photosensitiser, and thus the treated tumour volume, may therefore be maximised.

Tumour damage by PDT occurs either by direct tumour cell killing or, more importantly, by secondary cell killing effects induced after severe vascular damage and ischaemia (Henderson *et al.*, 1985). This phenomenon of vascular damage and induced hypoxia (Moore *et al.*, 1993) occurring at very short intervals following treatment led to the use of PDT in conjunction with bioreductive drugs, which are specifically toxic to hypoxic cells. Such studies have demonstrated that PDT in combination with bioreductive drugs can give enhanced anti-tumour effect (Gonzalez *et al.*, 1986; Bremner *et al.*, 1992; Baas *et al.*, 1994).

The purpose of this study was to measure the influence of *m*-THPC- or BCA-mediated PDT on the vascular perfusion and tumour response (regrowth time, cures and *in vitro* cell survival) in subcutaneous RIF1 tumours after *in vivo* PDT and to compare these results with Photofrin-mediated PDT. The vascular perfusion data were also required as a rational basis for the development of protocols involving combined PDT with the bioreductive agent mitomycin C (MMC).

Correspondence: FA Stewart, Department of Experimental Therapy (H6), The Netherlands Cancer Institute/Antoni van Leeuwenhoekhuis, Plesmanlaan 121, 1066 CX Amsterdam, The Netherlands
Received 17 January 1995; revised 15 March 1995; accepted 16 March 1995

### Materials and methods

### *Animal models*

All experiments were carried out in accordance with protocols approved by the local experimental animal welfare committee and conform to national and European regulations for animal experimentation. Female C3H/Km mice were used in all experiments, weighing 21-30 g at an age of 11-16 weeks. Approximately $1 \times 10^5$ RIF1 cells (maintained and passaged according to recommended *in vivo/in vitro* protocols described by Twentyman *et al.*, 1980) were inoculated subcutaneously on the lower dorsum of mice, which were briefly anaesthetised with enflurane. Tumour growth was documented three times weekly by calliper measurements in three orthogonal diameters. PDT was given 10-21 days after inoculation when the tumour reached a diameter of 5-6 mm. The tumours were free of evident necrosis at these sizes. The tumour response to PDT was determined by the number of cures or by regrowth time. Cures were defined as no visible or palpable evidence of tumour at 90 days. Tumour regrowth time was calculated as the time taken to regrow from treatment size $T$ to mean geometric diameter $T + 2$ mm. Mice were sacrificed when tumours had reached a mean diameter of $>10$ mm. A minimum of six mice per dose group were treated.

### *Interstitial photodynamic therapy of tumours*

The mice were injected i.v. with *m*-THPC (supplied by Scotia Pharmaceuticals, UK) or BCA (synthesised, purified and supplied by Dr Schuitmaker; Schuitmaker *et al.*, 1990). *m*-THPC was dissolved in 30% polyethylene glycol 400 (PEG), 20% ethanol and 50% water at a concentration of 0.03-0.06

mg ml$^{-1}$ (for doses of 0.15–0.3 mg kg$^{-1}$). BCA was dissolved in 3 ml of methanol: 10% Cremophor EL (Sigma, St Louis, MO, USA) and 3% 1,2-propanediol were added. Thereafter the methanol was evaporated under reduced pressure and finally the mixture was diluted with sterile 0.9% saline. The final concentration was 4.5 mg ml$^{-1}$ and doses of 20 mg kg$^{-1}$ were administered. Photofrin (supplied by Quadra Logic Technologies, Vancouver, Canada) was dissolved in 5% dextrose at a concentration of 2 mg ml$^{-1}$ for a dose of 10 mg kg$^{-1}$. Photofrin was injected i.p. for the estimates of cell survival after *in vivo* PDT, since previous experiments for tumour response studies were carried out with i.p. injections. However, no difference in tumour response was seen for Photofrin (10 mg kg$^{-1}$) administered i.v. or i.p. at 1 day before illumination (Van Geel *et al.*, 1995). The photosensitisers were injected 15 min (BCA), 1 h (BCA and *m*-THPC) or 1 day (*m*-THPC and Photofrin) before illumination; time intervals were chosen based on previous studies indicating times of maximum drug uptake and photosensitisation (Table I). Separate groups of control mice received BCA or *m*-THPC alone (Table II). The bioreductive drug mitomycin C (Kyowa, Japan) was administered in doses which caused minimal acute toxicity, i.e. less than 5% weight loss. The drug was dissolved in sterile water to a concentration of 0.5 mg ml$^{-1}$. Doses of 5 mg kg$^{-1}$ were given i.p. 15 min before illumination, or immediately after illumination; control groups receiving MMC alone, MMC in combination with BCA or *m*-THPC or MMC plus illumination were also included (Table II).

For *m*-THPC and Photofrin PDT, the light source was a dye laser (Spectra Physics model 373) pumped by a 12 W argon laser (Spectra Physics model 171). Sulphorhodamine B (Radiant Dyes Chemie, Wermelkirchen, Germany) was used as the dye to obtain red laser light (mono Chromator Oriel model 77320) of 628 ± 3 nm (Photofrin) or 652 ± 3 nm (*m*-THPC). The light was directed to a beam splitter that equally divided the light among four outputs, to which non-scintillating polystyrene fibres (Bicron BCF, 1 mm outer diameter) with 1 cm cylindrical diffusing tips were attached. The output from each fibre was adjusted to 100 mW cm$^{-1}$ and energies of 15–180 J cm$^{-1}$ were delivered, by varying the exposure time from 2.5 to 30 min, at 1 h or 1 day after mTHPC. Light alone controls were also treated (Table II). For tumour illuminations, the diffusing fibre tips were inserted through the centre of the tumours of unanaesthetised mice held in restraining jigs, as described by Baas *et al.* (1993).

For BCA-PDT a pigtailed (fibre diameter 100 μm multimode) diode laser (Philips), lasing at 750 nm maximum (output 200 mW), was used (Best *et al.*, 1993). Light from the diode laser was coupled into a 400 μm multimode fibre with 1 cm cylindrical diffusing tip (1.25 mm outer diameter, QLT Phototherapeutics, NY, USA). This multimode fibre was used because the coupling of this fibre was more efficient for this experimental set-up than coupling of a non-scintillating polystyrene fibre. Light doses of 0–400 J cm$^{-1}$ were delivered at 15 min or 1 h after BCA. Light-alone controls were also included at the 750 nm wavelength (Table II). Mice were kept in subdued light for 2 weeks after receiving the photosensitisers.

### *$^{86}$RbCl extraction estimates for vascular perfusion*

Vascular perfusion relative to the cardiac output was measured using the $^{86}$RbCl extraction technique (Van Geel *et al.*, 1994). Each mouse was injected via the tail vein at 5 min to 5 days after PDT with 0.1 ml of a rubidium chloride solution (specific activity 1–8 mCi mg$^{-1}$, from Amersham, Aylesbury, UK), which had been diluted with saline (0.9%) to an activity of approximately 100 μCi ml$^{-1}$. After 1 min, the mice were killed by cervical dislocation. The tumour, kidney (control organ) and the tail were then removed and the weighed samples counted in a gamma counter (Packard Delft, The Netherlands, Minaxi Autogamma 5000 series) for 50 min (according to the expected level of activity) together with a 0.1 ml aliquot of the injection solution which was used as a standard. Four to six mice per group were used for these experiments. The tails were excised to check the residual activity at the site of the injection (5–10% of the injected dose in most mice) and the sample counts were corrected for radioactivity in the tail before calculating the percentage of injected dose in tumour or kidney. If the residual activity in the tail was 15% or more, the samples were excluded from analysis. Control groups receiving sensitiser alone, light alone (652 and 750 nm) or no treatment were also tested for tumour perfusion (Table III).

**Table I** Photosensitisation and tumour uptake after injection of Photofrin, *m*-THPC and BCA

| | *TI*[a] *(h)* | *Tumour uptake* | *Regrowth time (days)*[b]*/light dose (J cm$^{-1}$)* |
|---|---|---|---|
| | | μg g$^{-1}$ [c] | |
| Photofrin | 6 | 2.7 ± 0.3 | 16.7 ± 1.5/200 |
| | 24 | 3.0 ± 0.3 | 16.0 ± 0.9/200 |
| | 72 | 2.4 ± 0.5 | 15.7 ± 0.9/240 |
| | | *% inj g$^{-1}$* [d] | |
| *m*-THPC | 1 | 0.5 ± 0.1 | [e]/150 |
| | 24 | 0.8 ± 0.2 | 22.9 ± 2.1/150 |
| | 72 | 1.1 ± 0.4 | 9.1 ± 0.4/150 |
| | | *Fluorescence*[f] | |
| BCA | 0.25 | ~125 | 17.5 ± 5.6/150 |
| | 1 | ~120 | 9.8 ± 1.4/150 |
| | 2 | ~80 | –/150 |

[a]TI, time interval between photosensitiser and illumination. [b]10 mg kg$^{-1}$ Photofrin (Baas *et al.*, 1994); 0.15 mg kg$^{-1}$ *m*-THPC (Van Geel *et al.*, 1995) and 20 mg kg$^{-1}$ BCA (present results). [c]Spectrofluorometry measurements; RIF1 mouse tumours (Van Geel *et al.*, 1994). [d][$^{14}$C]*m*-THPC uptake in RIF1 mouse tumours (Van Geel *et al.*, unpublished). [e]Lethal toxicity. [f]Fluorescence measurements based on image analysis of grey scale values 0–200; RMA rat tumours (Van Leengoed *et al.*, 1993).

**Table II** Tumour regrowth times in the control groups

| *Treatment* | *Regrowth time (days)* |
|---|---|
| Untreated control | 2.8 ± 0.1 |
| *m*-THPC (0.3 mg kg$^{-1}$) | 4.5 ± 0.5[a] |
| BCA (30 mg kg$^{-1}$) | 4.4 ± 0.3[a] |
| MMC (5 mg kg$^{-1}$) | 7.3 ± 0.3[a] |
| *m*-THPC (0.3 mg kg$^{-1}$) + MMC (5 mg kg$^{-1}$) | 7.2 ± 0.7[a] |
| BCA (20 mg kg$^{-1}$) + MMC (5 mg kg$^{-1}$) | 6.4 ± 0.6[a] |
| Light alone (λ = 652 nm: 60 J cm$^{-1}$) | 3.8 ± 0.2[a] |
| Light alone (λ = 750 nm: 300 J cm$^{-1}$) | 4.5 ± 0.5[a] |
| MMC + light (λ = 652 nm: 60 J cm$^{-1}$) | 7.6 ± 0.8[a] |
| MMC + light (λ = 750 nm: 200 J cm$^{-1}$) | 5.0 ± 0.3[a] |

[a]Significantly different from untreated control group ($P < 0.01$).

### In vivo/in vitro *assay*

Tumours treated with Photofrin PDT or *m*-THPC PDT and control tumours treated with photosensitiser alone, light alone, MMC alone or receiving no treatment (Table IV) were excised under sterile conditions after cervical dislocation. Two tumours were pooled for each time point. For BCA PDT no *in vivo/in vitro* assays were performed because with this experimental set-up only one mouse could be treated per time point. The excised tumours were protected from direct light exposure, weighed, minced with scissors and placed into a flask containing protease (type IX), DNAse (type I) and collagenase (type IV) in 5 ml of sterile phosphate-buffered saline (PBS) and stirred at 37°C for 30 min to release tumour cells. The resulting suspension was then strained through a wire mesh to eliminate any remaining tissue clumps and cell aggregates. The cells were washed with Ham's F-10 medium containing antibiotics (penicillin 100 IU ml$^{-1}$ and streptomycin 100 μg ml$^{-1}$) without serum and centrifuged for 5 min at 1000 r.p.m. The supernatant was discarded and the cell pellet resuspended in 10 ml of Ham's F-10 medium containing antibiotics and 10% fetal calf serum. Cell numbers were determined with a Coulter counter, and after staining with trypan blue the percentage of dead cells was counted with a haemocytometer. Known numbers of viable cells were plated into Petri dishes at appropriate concentrations in Ham's F-10 medium containing antibiotics and 10% fetal calf serum for colony formation. After 6 days in a humidified 5% carbon dioxide incubator at 37°C, the cells were washed with saline (0.9%) and fixed and stained with glutaraldehyde (15%, w/w) and crystal violet (2%, w/w). Colonies containing >50 cells were then counted. The Coulter counter and haemocytometer counts yielded information from which viable cell yield per gram of tumour was calculated on the basis of total cell counts. Colony assays were used to determine the fraction of viable cells which were clonogenic. The combination was used to calculate the yield of surviving cells per gram of tumour.

### *Statistical analysis*

Means and standard error of the means (s.e.m.) were calculated for tumour regrowth times for each treatment group. The cures were not included in the estimates of mean regrowth time but were analysed separately. The $TCD_{50}$ and standard errors (± s.e.) (light dose required to cure 50% tumours) were calculated by probit analysis. For the *in vivo*/*in vitro* studies mean and s.e. were calculated.

The significance of difference in vascular perfusion and clonogenicities for the control groups and treated groups was determined according to the non-parametric Kruskal–Wallis test; *P*- values of ⩽0.05 were considered significant. Kendal-l's tau correlation coefficient between vascular perfusion and tumour regrowth time was calculated from the group means for each photosensitiser; cured tumours were also taken into account in assessing significance.

The association of the different treatments with the regrowth time was analysed using non-parametric survival analysis methods. To study simultaneously the influence of photosensitiser, light and MMC on regrowth time, a Wilcoxon's proportional hazard model was adjusted using a stepwise procedure. The Breslow test (generalised Wilcoxon test) was applied to compare PDT with or without MMC. The statistical package BMDP module 2L was used.

### Results

The tumour perfusion data are analysed as $^{86}$Rb counts per gram of tissue, expressed as a percentage of the injected activity (minus residual activity in the tail resulting from leakage at the injection site) (Figure 1 and Table III). This gives a measure of the proportion of the cardiac output supplying that tissue. Light-alone (fibre insertion with light

**Figure 1** (**a**) Relative perfusion of the RIF1 tumours 5 min after illumination with different light doses given 24 h after 0.15 (●) and 0.3 (▼) mg kg$^{-1}$ *m*-THPC or 15 min after 20 mg kg$^{-1}$ BCA (□). (**b**) Effect of PDT (30 J cm$^{-1}$) at 24 h after 0.15 (●) and 0.3 (▼) mg kg$^{-1}$ *m*-THPC or 15 min after 20 mg kg$^{-1}$ BCA (□) on relative perfusion at increasing times after illumination. Control values (shown at 0 J cm$^{-1}$ light dose) represent drug alone tumour perfusion. Perfusion after Photofrin PDT (previously published results; Van Geel *et al*., 1994) are also shown with the dashed lines. Values are mean ± s.e.m. of 4–6 mice per group.

**Table III** Vascular perfusion (% g$^{-1}$ tissue) in control groups of tumours or kidneys

| *Treatment* | *Tumour* | *Kidney* |
|---|---|---|
| Untreated control | 2.2 ± 0.2 | 23.5 ± 3.2 |
| *m*-THPC (0.3 mg kg$^{-1}$) | 1.9 ± 0.2 | 20.4 ± 1.0 |
| BCA (20 mg kg$^{-1}$) | 1.8 ± 0.2 | 24.6 ± 2.6 |
| Light alone (λ = 652 nm: 30 J cm$^{-1}$) | 1.9 ± 0.3 | 30.3 ± 2.5[a] |
| Light alone (λ = 750 nm: 200 J cm$^{-1}$) | 0.8 ± 0.2[b] | 19.9 ± 2.6 |

[a]Significantly different from control group ($P<0.05$). [b]Significantly different from control group ($P<0.02$).

**Table IV** Tumour survival parameters control groups

| *Treatment* | *Viable cells g$^{-1}$ tumour tissue*[a] *(× 10$^7$)* | *Percent control* | *Plating efficiency*[a] *(%)* | *Percent control* | *Clonogenicity (clonogenic cells g$^{-1}$ tumour)*[a,b] *(× 10$^7$)* | *Surviving fraction (%)* |
|---|---|---|---|---|---|---|
| Untreated control | 15.7 ± 2.4[c] | 100 | 33.8 ± 4.3 | 100 | 5.4 ± 1.35 | 100 |
| Fibre control | 7.7 ± 2.6 | 49 | 21.5 ± 2.4 | 64 | 1.6 ± 0.49 | 30 |
| Light control (λ = 630 nm) | 9.4 ± 0.6 | 60 | 25.4 ± 4.2 | 75 | 2.4 ± 0.45 | 44 |
| Light control (λ = 652 nm) | 9.8 ± 1.1 | 62 | 24.1 ± 2.3 | 71 | 2.4 ± 0.47 | 44 |
| *m*-THPC (0.15 mg kg$^{-1}$) | 9.1 ± 1.7 | 58 | 27.1 ± 6.1 | 80 | 2.8 ± 0.96 | 52 |
| Photofrin (10 mg kg$^{-1}$) | 11 ± 2.1 | 70 | 36.8 ± 5.0 | 109 | 3.6 ± 0.47 | 67 |
| MMC alone (24 h)[d] | 7.4 ± 2.6 | 47 | 0.4 ± 0.2[d] | 1 | 0.04 ± 0.02[e] | 0.7 |

[a]Means ± s.e.m. [b]Cell yield × plating efficiency. [c]The percentage of dead cells stained with trypan blue was 5%. [d]Mice were sacrificed 24 h after MMC injection. [e]Significantly different from control group ($P<0.05$).

treatment) and drug-alone controls were also included for both sensitisers. Both m-THPC and BCA alone caused small non-significant decreases in tumour perfusion. Illumination with 30 J cm$^{-1}$ 652 nm light also caused a small, non-significant decrease in tumour perfusion. The vascular perfusion did decrease significantly after light alone with the illumination wavelength and set-up used for the BCA experiments, probably caused by the larger outer diameter of the fibre tip used for these illuminations. m-THPC PDT and BCA PDT caused a light dose-dependent reduction in perfusion which was very similar to that seen after Photofrin-PDT (Van Geel *et al.*, 1993) (Figure 1a). For m-THPC (0.15 and 0.3 mg kg$^{-1}$) and BCA PDT the vascular perfusion decreased significantly from approximately 1.8% of the injected dose in untreated tumours to ± 0.5% at 5 min after 100–120 J cm$^{-1}$. Subsequent experiments investigated the influence of PDT on tumour perfusion at longer time intervals of up to 5 days after 30 J cm$^{-1}$ illumination (Figure 1b). The minimum perfusion was at 24 h for all photosensitisers, but with m-THPC and BCA the decrease in perfusion from 5 min to 24 h was more pronounced than for Photofrin. No vascular perfusion could be measured beyond 24 h after BCA or 0.3 mg kg$^{-1}$ m-THPC since the tumorous tissue had been destroyed. For Photofrin PDT at least 100 J cm$^{-1}$ was required for the same decrease in vascular perfusion as seen with BCA or m-THPC with 30 J cm$^{-1}$ (Van Geel *et al.*, 1994).

The measured values for mean tumour perfusion at 5 min after PDT were compared with the mean regrowth time achieved for the same drug and light treatments (separate experiments). A light dose range of 15–120 J cm$^{-1}$ or 30–400 J cm$^{-1}$ was used for tumour regrowth studies for m-THPC PDT and BCA PDT respectively. The Kendall's tau correlation coefficient between tumour growth time and perfusion was $R = -0.80$, $-0.60$, $-0.73$ and $-0.67$ respectively for Photofrin (Van Geel *et al.*, 1994) m-THPC (0.15 mg kg$^{-1}$), m-THPC (0.3 mg kg$^{-1}$) and BCA (20 mg kg$^{-1}$). For all tested photosensitisers there was an inverse linear relationship between tumour regrowth time and vascular perfusion, but for m-THPC and BCA the correlation was less strong.

### In vitro *tumour cell survival after* in vivo *PDT*

The correlation of vascular perfusion and regrowth time suggested that for m-THPC a decrease in vascular perfusion seemed to be less important for tumour response than for Photofrin. Direct PDT-induced tumour cell death may therefore be more important for m-THPC than indirect vascular effects. This hypothesis was tested in an *in vivo/in vitro* assay in which the time course of cell death was determined by progressively delaying the time of tumour excision after *in vivo* treatment. For each experiment, three parameters were recorded: total cell yield per gram of tumour tissue; plating efficiency of recovered cells; and the product of cell yield and plating efficiency, which defines the number of surviving clonogens per gram of tumour. Light and drug doses were chosen which resulted in similar regrowth times for the different photosensitisers (0.15 mg kg$^{-1}$ m-THPC with 60 J cm$^{-1}$ gives 15.3 ± 1.2 days regrowth time; or 10 mg kg$^{-1}$ Photofrin with 150 J cm$^{-1}$ gives 14.6 ± 1.3 days regrowth time).

Table IV shows the tumour survival parameters for various control groups. The percentage of dead cells after trypan blue staining was 5% for the untreated control group. The number of clonogens per gram of tumour fell to 30–44% of untreated control values as a result of fibre insertion with or without illumination. m-THPC or Photofrin alone reduced the clonogens g$^{-1}$ to 52% and 67% of the control values respectively. When m-THPC PDT or Photofrin PDT was administered and tumour cell suspensions were made immediately after treatment, a further reduction in cell yield and plating efficiency was observed (Figure 2). This probably resulted from direct cell death caused by PDT. There was no significant difference in plating efficiency between the two

**Figure 2** Tumour cell survival kinetics following *in vivo* PDT as a function of excision time. Photofrin PDT: 10 mg kg$^{-1}$, 150 J cm$^{-1}$ (open symbols). m-THPC PDT: 0.15 mg kg$^{-1}$, 60 J cm$^{-1}$ (closed symbols). Squares: plating efficiency. Circles: yield of viable cells$^{-1}$ g$^{-1}$ tumour tissue. Triangles: clonogenic cells$^{-1}$ g$^{-1}$ tumour. Data points represent group mean values ± s.e.

photosensitisers, but a small difference was found in cell yield when the tumours were excised 1, 6 and 16 h after illumination, resulting in more clonogenic cells g$^{-1}$ tumour for m-THPC. Excision of the tumours after 24 h showed similar decreases in cell yield to 1% and plating efficiency to 40% for both photosensitisers.

It was clear from tumour perfusion experiments that all three photosensitisers induced a substantial reduction in perfusion for subcurative light doses. It has been suggested that transient PDT-induced hypoxia could be exploited to enhance the tumoricidal effects of bioreductive drugs which are specifically toxic against hypoxic cells (Gonzalez *et al.*, 1986). The effect of Photofrin or m-THPC PDT in combination with MMC was therefore also tested in the *in vivo/in vitro* experiments. MMC alone caused no more than a 4 day delay in tumour regrowth *in vivo* and reduced the surviving fraction to <1% when assayed by the *in vivo–in vitro* technique 24 h after injection (Table V). Other experiments with MMC alone showed a 0.34% ± 0.24% surviving fraction when assayed 20 min after MMC injection. The unexpectedly low surviving fractions, in relation to the modest 4 days tumour regrowth time (Table II), may have been due to the drug being carried over with the tumour into the dispersal medium and causing additional cell killing (Twentyman, 1977). Untreated and MMC-treated tumour halves were therefore combined and tested for this. The killing artifact was apparently absent here, since the plating efficiency of the mixture could be adequately predicted from the separate plating efficiencies without invoking interactions. An alternative explanation is that cells are made sensitive to the killing action of MMC during dispersal (the number of living cells decreased from 93% control value to about 80%).

For Photofrin and m-THPC PDT in combination with MMC, the 24 h time point is reliable since the half-life of MMC is 10–50 min under physiological conditions, therefore no free drug will be present at this time. Cell survival assessed 24 h after equitoxic drug light schedules (15 days regrowth time), was significantly lower after PDT in combination with MMC than after PDT alone. Clonogenicities for Photofrin and m-THPC PDT in combination with MMC were 0.02% ± 0.003% and 0.3% ± 0.07% respectively. These values are consistent with additive cell killing from MMC and PDT.

*Tumour regrowth studies after PDT in combination with MMC*

Control experiments were performed to evaluate the individual effects of photosensitisers, MMC, light or fibre insertion alone on tumour regrowth (Table II). A small but significant increase in growth time occurred when the photosensitiser, fibre and/or light alone was given. MMC alone also induced a small, but significant, increase in tumour regrowth time. The combination of MMC with *m*-THPC, BCA or illumination (652 nm) was not significantly different from MMC alone.

BCA was tested in tumour regrowth studies in a concentration of 10–30 mg $kg^{-1}$, given 15 min or 1 h before light treatment (Figure 3). When BCA was given 15 min before illumination, regrowth time was greater than with the 1 h time interval. BCA in a concentration of 30 mg $kg^{-1}$ given 15 min before light treatment was too toxic (1/3 deaths). The schedule of 20 mg $kg^{-1}$ given at 15 min before illumination was therefore chosen for further studies. Figure 4 shows the mean tumour regrowth times after the combined treatment of PDT and MMC compared with *m*-THPC or BCA PDT alone. The *in vivo* photosensitising ability of both drugs was compared with previously published results for Photofrin PDT with or without MMC (Baas *et al.*, 1994; dashed lines in Figure 4). *m*-THPC-mediated PDT alone (Van Geel *et al.*, 1995) resulted in longer regrowth times and more cures than Photofrin-mediated PDT, at much lower light doses (Figure 4, Table V). Reducing the time interval between *m*-THPC and illumination from 1 day to 1 h did not increase tumour growth delay (Figure 4), but more cures were seen at lower

**Figure 3** Tumour regrowth time for increasing doses of BCA (10–30 mg $kg^{-1}$) given 15 min (□) or 1 h (■) before illumination with 150 J $cm^{-1}$. For the 30 mg $kg^{-1}$ drug dose, one out of three mice died at the 15 min interval (†). For the other groups there were no deaths and values are mean tumour regrowth times ± s.e.m. of six mice per group. Groups containing animals with cured tumours are indicated by asterisks.

**Figure 4** Regrowth time of the RIF1 tumour after PDT with (closed symbols) or without MMC (open symbols) with increasing light doses. *m*-THPC in the concentration 0.15 mg $kg^{-1}$ was given 1 h (◇) or 1 day (○) before illumination. *m*-THPC in a concentration of 0.3 mg $kg^{-1}$ was given 1 day before illumination and BCA in a concentration of 20 mg $kg^{-1}$ 15 min before illumination. Previously published Photofrin data (Baas *et al.*, 1994) for regrowth time after PDT alone (– – –) or with MMC (——) are also indicated by the dashed lines. The data for *m*-THPC PDT were previously published (Van Geel *et al.*, 1994). Values are mean ± s.e.m. of 6–10 mice per group. When no error bars are visible they were smaller than the symbol.

**Table V** Number of cures per treatment group: photosensitiser was given 15 min, 1 h or 1 day before illumination

| *Light dose* ($J\ cm^{-1}$) | m-*THPC (1 h)* *0.15 mg* $kg^{-1}$ *– MMC*[a] | *+ MMC* | m-*THPC (1 day)* *0.15 mg* $kg^{-1}$ *– MMC*[a] | *+ MMC* | m-*THPC (1 day)* *0.3 mg* $kg^{-1}$ *– MMC*[a] | *+ MMC* | *BCA (15 min)* *20 mg* $kg^{-1}$ *– MMC* | *+ MMC* |
|---|---|---|---|---|---|---|---|---|
| 0 | 0/8 | 0/8 | 0/8 | 0/17 | 0/14 | 0/15 | 0/6 | |
| 15 | – | – | – | – | 0/8 | – | – | – |
| 30 | 0/8 | 3/12 | 0/8 | 1/8 | 0/8 | 0/8 | 0/6 | 0/6 |
| 45 | – | 5/10 | – | – | – | 1/8 | – | – |
| 60 | 1/8 | 2/10 | 0/8 | 2/8 | 0/8 | 3/8 | – | – |
| 75 | – | – | – | – | – | – | – | – |
| 90 | 6/7[b] | – | 3/8 | 3/8 | 4/8 | 7/8 | – | – |
| 100 | – | – | – | – | – | – | 0/6 | 0/6 |
| 120 | 5/9 | – | 1/8 | 4/8 | 3/8[b] | – | – | – |
| 150 | [c] | – | 0/8 | – | – | – | 1/5 | – |
| 180 | – | – | 1/8 | – | – | – | – | – |
| 200 | – | – | – | – | – | – | 5/6 | 1/6 |
| 240 | – | – | – | – | – | – | – | – |
| 300 | – | – | – | – | – | – | 3/6 | – |
| 400 | – | – | – | – | – | – | 2/5[b] | – |
| $TCD_{50}$[d] ($J\ cm^{-1}$) | 93 ± 12 | | | 103.7 ± 18.5 | 124 ± 25 | 66.5 ± 6.2 | | |

[a]Previously published (Van Geel *et al.*, 1995). [b]12.5–20% deaths were found in these groups: only survivors are given in the denominator. [c]<12.5% survivals. [d]Light dose required to cure 50% of the tumours. –, not determined.

light doses (Table V). This time interval was, however, also more toxic and led to deaths in combination with high light doses. BCA PDT gave approximately the same tumour regrowth delay as Photofrin PDT, but cures were found at light doses of 150 J $cm^{-1}$, whereas for Photofrin PDT no cures were found below 400 J $cm^{-1}$ (Table III).

After PDT alone, a plateau in regrowth time was reached at 15–20 days. Some cures were found at higher light doses, but there was no further increase in regrowth time. This may be indicative of a PDT-resistant cell population, and PDT in combination with MMC may overcome this problem. *m*-THPC PDT in combination with MMC resulted in highly significant increases in tumour response compared with PDT alone for both drug concentrations and all light doses tested for the 1 h and 1 day interval. MMC given immediately after illumination (*m*-THPC PDT) was equally effective (24.5 ± 1.71 days regrowth time, 5/8 cures) as MMC given 15 min before illumination (25.6 ± 1.69 days regrowth time, 3/8 cures). The light dose for 50% cures ($TCD_{50}$) decreased from 124 ± 25 J $cm^{-1}$ at 1 day after 0.3 mg $kg^{-1}$ *m*-THPC to 67 ± 6J $cm^{-1}$ for 0.3 mg $kg^{-1}$ in combination with MMC. No $TCD_{50}$ could be determined for 0.15 mg $kg^{-1}$ *m*-THPC 1 day before illumination, but for the combination mTHPC (0.15 mg $kg^{-1}$) with MMC the $TCD_{50}$ was 104 ± 19 J $cm^{-1}$. The combination BCA PDT with MMC did not result in a significant increase in tumour regrowth time or more cures compared with BCA PDT alone when the sensitiser was given 15 min before illumination. When BCA was injected 1 h before illumination (100 J $cm^{-1}$), however, the combination with MMC resulted in a significant increase in regrowth time (from 12.9 ± 1.1 to 23.1 ± 2.9 days). No $TCD_{50}$ could be calculated for BCA PDT with or without MMC. Previously published results have demonstrated that the $TCD_{50}$ for Photofrin-mediated PDT decreases from 731 ± 70 J $cm^{-1}$ for PDT alone to 319 ± 49 J $cm^{-1}$ when Photofrin PDT is given in combination with MMC (Baas *et al.*, 1994).

### Discussion

PDT can cause cell death both by direct disruption of the cellular membranes and organelles, e.g. mitochondria (Grossweiner, 1984), and by damage to the tumour vasculature leading to secondary tumour cell death (Castellani *et al.*, 1963; Bugelski *et al.*, 1981; Star *et al.*, 1986). Using the $^{86}Rb$ technique (Van Geel *et al.*, 1994), we have shown that non-curative doses of *m*-THPC and BCA PDT decrease vascular perfusion in the RIF1 tumour for a period of at least 24 h. This decrease was more pronounced for *m*-THPC and BCA PDT than for Photofrin PDT for the same light dose. In previous experiments (Van Geel *et al.*, 1994) we found an inverse relationship between vascular perfusion after sub-curative Photofrin PDT and regrowth time in the RIF1 tumour. An inverse correlation was also found for *m*-THPC and BCA PDT, but the correlation was much less pronounced possibly indicating a greater component of direct tumour cell killing for these sensitisers.

With *in vivo*/*in vitro* experiments we have tried to separate the direct tumour cell killing (excision immediately after treatment) from indirect vascular effects (delayed excision and assessment of clonogenicity) as previously described by Henderson *et al.* (1985). Delivery of *in vivo* PDT treatment did not immediately lead to a reduction in tumour clonogenicity for either *m*-THPC or Photofrin, implying that the phototoxic effect on the tumour cells was not sufficient to render them non-reproductive. It was necessary for the tumours to remain *in situ* after completion of treatment for tumour cell death to occur. Similar observations were made by Henderson *et al.* (1985), who found that when cells were excised and plated immediately after Photofrin PDT there was no reduction of cell survival *in vitro*, although *in vivo* tumour necrosis was observed after the doses used. When the cells were left *in situ* for 24 h and then plated, the *in vitro* survival was markedly reduced. The authors interpreted this effect as evidence of delayed secondary tumour cell death due to vascular damage and induced hypoxia, with little direct toxicity. Our results indicated that clonogenicity decreases were slightly, but not significantly, slower after *m*-THPC PDT than for Photofrin. These findings do not support the original hypothesis of greater direct tumour cell killing after *m*-THPC PDT. This suggests that tumour destruction *in vivo* involves additional factors other than direct tumour cell killing or damage to the vasculature, e.g. host-related factors.

A potential way of improving the tumoricidal effects of PDT is to exploit the induced chronic hypoxia by combining PDT with bioreductive drugs. Since bioreductive drugs are activated to a cytotoxic product under hypoxic conditions, their activity can be enhanced *in vivo* when used together with treatments that enhance the depth or duration of hypoxia in solid tumours. The first reports of PDT combined with bioreductive drugs were from Gonzalez *et al.* (1986). They found that a large increase in the regrowth time of Dunning rat tumours was seen when misonidazole was administered 20 min before or after illumination. Several other investigators (Bremner *et al.*, 1992; Baas *et al.*, 1994) have subsequently demonstrated a clear advantage for the combined treatment of PDT plus various bioreductive drugs *vs.* PDT alone.

PDT in combination with MMC gave a significant decrease in the number of clonogens $g^{-1}$ tumour in our *in vivo*/*in vitro* experiments at 24 h after Photofrin- or *m*-THPC-mediated PDT, mainly caused by a large decrease in plating efficiency. *In vivo* tumour response experiments also demonstrated that MMC given 15 min before PDT substantially increased tumour regrowth time. Light doses could be reduced by a factor of 2 in combination with MMC for equivalent effects compared with PDT alone for *m*-THPC PDT. These results were similar to those previously reported for Photofrin-mediated PDT in combination with MMC. For *m*-THPC PDT, however, MMC given after illumination was equally as effective as MMC before illumination. This is different from the results found with Photofrin PDT (Baas *et al.*, 1994), where the maximum benefit from the combination MMC + PDT is obtained when the drug is given before illumination. This difference may be due to the higher light dose (400 J $cm^{-1}$) required for effective tumour treatment with Photofrin-mediated PDT. These light doses require 68 min for delivery, by which time vascular perfusion probably inhibits the access of MMC into the tumour (Van Geel *et al.*, 1994). For BCA PDT in combination with MMC, no increase in tumour response was found compared with BCA PDT alone when the sensitiser was administered at 15 min before illumination. This may be explained by the fact that MMC was injected at the same time as BCA. The dispersion of BCA through the tumour may not be optimal at such a short time interval, resulting in fewer BCA PDT-damaged tumour cells being triggered to die by the action of MMC. When the BCA was injected 1 h before illumination, MMC did increase the PDT response.

Our experiments demonstrate that there is a reduced vascular perfusion in the RIF1 tumour after various doses of *m*-THPC or BCA PDT, which persist for at least 24 h. No distinction could be made between *m*-THPC and Photofrin PDT when looking at direct tumour cell death and secondary cell death in our *in vivo*/*vitro* assay. Hypoxia induced by vascular damage can be exploited by the combination of *m*-THPC PDT and MMC. Enhancement of the tumoricidal effect means that lower drug and light doses can be used for equal tumour effects. The use of lower photosensitiser doses would reduce the skin photosensitisation associated with PDT in the clinic. The advantage of lower light doses is that treatment times are reduced, which is favourable particularly for less efficient sensitisers. This would enable a more widespread use of PDT, with treatment of larger surface areas within acceptable time limits.

**Acknowledgements**
We are grateful to Dr A Begg and Dr P Baas for many helpful discussions and to Dr O Dalesio for the statistical analysis. We thank Quadra Logic Technologies, Vancouver, Canada, for giving us Photofrin, and Scotia, Guildford, UK, for supplying us with *m*-THPC. The present work was supported by the Dutch Cancer Society, Project NKI 91-05.

**References**

BAAS P., OPPELAAR H, STAVENUITER M, ZANDWIJK N AND STEWART FA. (1993). Interaction of the bioreductive drug (SR4233) and photodynamic therapy using Photofrin II in a mouse tumour model. *Int. J. Radiat. Biol. Oncol. Phys.*, **27**, 665–670.

BAAS P, OPPELAAR H, MICHIELSEN C, ZANDWIJK N AND STEWART FA. (1994). Enhancement of interstitial photodynamic therapy by mitomycin C and EO9 in a mouse tumour model. *Int. J. Cancer*, **56**, 880–885.

BEST JA, SCHUITMAKER JJ, DUBBELMAN TMAR, VAN DER POEL CJ, FAKKEL J. (1993). Near infrared diode laser for photodynamic tumour therapy using BCA. *Lasers Med. Sci.*, **8**, 157–162.

BREMNER JCM, ADAMS GE, PEARSON JK, SANSOM J, STRATFORD IJ, BEDWELL J, BOWN SG AND PHILIPS D. (1992). Increasing the effect of photodynamic therapy on the RIF1 murine sarcoma, using the bioreductive drugs RSU1069 and RB6145. *Br. J. Cancer*, **66**, 1070–1076.

BUGELSKI PJ, PORTER CW AND DOUGHERTY TJ. (1981). Autoradiographic distribution of hematoporphyrin in normal and tumour tissue of the mouse. *Cancer Res.*, **41**, 4606–4612.

CASTELLANI A, PACE GP AND CONCIOLI M. (1963). Photodynamic effect of haematoporphyrin on blood microcirculation. *J. Pathol. Bacteriol.*, **86**, 99–102.

DOUGHERTY TJ, COOPER MT AND MANG TS. (1990). Cutaneous phototoxicity occurrences in patients receiving Photofrin. *Lasers Med. Surg.*, **10**, 485–488.

VAN GEEL IPJ, OPPELAAR H, OUSSOREN Y AND STEWART FA. (1994). Changes in perfusion of mouse tumours after photodynamic therapy. *Int. J. Cancer*, **56**, 224–228.

VAN GEEL IPJ, OPPELAAR H, OUSSOREN Y AND STEWART FA. (1995). Photosensitizing efficacy of mTHPC-PDT compared to Photofrin-PDT in the RIF1 mouse tumour and normal skin. *Int. J. Cancer*, **60**, 388–394.

GONZALEZ S, ARNFIELD MR, MEEKER BE, TULIP J, LAKEY WH AND CHAPMAN JD. (1986). Treatment of Dunning R3327-AT rat prostate tumours with photodynamic therapy in combination with misonidazole. *Cancer Res.*, **46**, 2858–2862.

GROSSWEINER LI. (1984). Membrane photosensitisation by hematoporphyrin and hematoporphyrin derivative. In *Photofrin Localization and Treatments of Tumours*, Gomer CJ and Doraian DR. (eds) pp. 391–404. Alan R. Liss: New York.

HENDERSON BW, STEPHEN MW, MANG TS, POTTER WR, MALONE PB AND DOUGHERTY TJ. (1985). Tumour destruction and kinetics of tumour cell death in two experimental mouse tumors following photodynamic therapy. *Cancer Res.*, **45**, 572–576.

VAN LEENGOED HLLM, SCHUITMAKER JJ, VAN DER VEEN N, DUBBELMAN TMAR, STAR WM. (1993). Fluorescence and photodynamic effects of bacteriochlorin a observed *in vivo* in 'sandwich' observation chambers. *Br. J. Cancer*, **67**, 898–903.

MOORE RB, CHAPMAN JD, MERCER JR, MANNAN RH, WIEBE LI, MCEWAN AJ, MCPHEE MS. (1993). Measurement of PDT-induced hypoxia in Dunning prostate tumors by iodine-123-iodoazomycin arabinoside. *J. Nucl. Med.*, **34**, 405–413.

RIS HB, ALTERMATT HJ, INDERBITZI R, NACHBUR B, STEWART JCM, WANG Q, LIM CK, BONNET R, BERENBAUM MC AND ALTHAUS U. (1991). Photodynamic therapy with chlorins for diffuse malignant mesothelioma: initial clinical results. *Br. J. Cancer*, **64**, 1116–1120.

RIS HB, ALTERMATT HJ, NACHBUR B, STEWART CM, WANG Q, LIM CK, BONNET R AND ALTHAUS U. (1993). Effect of drug-light interval on photodynamic therapy with metatetrahydroxyphenylchlorin in malignant mesothelioma. *Int. J. Cancer*, **53**, 141–146.

SCHUITMAKER JJ, BEST JA, DELFT JL, DUBBELMAN TMAR, OOSTERHUIS JA AND WOLFF-ROUENDAAL D. (1990). Bacteriochlorin a, a new photosensitizer in photodynamic therapy: In vivo results. *Invest. Opthalmol. Vis. Sci.*, **31**, 1444–1450.

STAR WM, MARIJNISSEN HPA, BERG-BLOK AE, VERSTEEG JAC, FRANKEN KAP AND REINHOLD HS. (1986). Destruction of rat mammary tumour and normal tissue microcirculation by hematoporphyrin derivative photoirradiation observed in vivo in sandwich observation chambers. *Cancer Res.*, **46**, 2532–2540.

TWENTYMAN PR. (1977). An artefact in clonogenic assays of bleomycin cytotoxicity. *Br. J. Cancer*, **36**, 642–644.

TWENTYMAN PR, BROWN JM, GRAY JW, FRANKA AJ, SCOLES MA AND KALLMAN RF. (1980). A new mouse tumour model system (RIF-1) for comparison of end-point studies. *J. Natl. Cancer Inst.*, **64**, 594–604.